# Bismuth atom tailoring of indium oxide surface frustrated Lewis pairs boosts heterogeneous CO_2_ photocatalytic hydrogenation

**DOI:** 10.1038/s41467-020-19997-y

**Published:** 2020-11-30

**Authors:** Tingjiang Yan, Na Li, Linlin Wang, Weiguang Ran, Paul N. Duchesne, Lili Wan, Nhat Truong Nguyen, Lu Wang, Meikun Xia, Geoffrey A. Ozin

**Affiliations:** 1 The Key Laboratory of Life-Organic Analysis, College of Chemistry and Chemical Engineering, Qufu Normal University, 273165 Qufu, Shandong People’s Republic of China; 2 Materials Chemistry and Nanochemistry Research Group, Solar Fuels Cluster, Department of Chemistry, University of Toronto, 80 St. George Street, Toronto, ON M5S 3H6 Canada; 3 Qufu Normal University Library, Qufu Normal University, 273165 Qufu, Shandong People’s Republic of China

**Keywords:** Photocatalysis, Solar fuels, Synthesis and processing

## Abstract

The surface frustrated Lewis pairs (SFLPs) on defect-laden metal oxides provide catalytic sites to activate H_2_ and CO_2_ molecules and enable efficient gas-phase CO_2_ photocatalysis. Lattice engineering of metal oxides provides a useful strategy to tailor the reactivity of SFLPs. Herein, a one-step solvothermal synthesis is developed that enables isomorphic replacement of Lewis acidic site In^3+^ ions in In_2_O_3_ by single-site Bi^3+^ ions, thereby enhancing the propensity to activate CO_2_ molecules. The so-formed Bi_x_In_2-x_O_3_ materials prove to be three orders of magnitude more photoactive for the reverse water gas shift reaction than In_2_O_3_ itself, while also exhibiting notable photoactivity towards methanol production. The increased solar absorption efficiency and efficient charge-separation and transfer of Bi_x_In_2-x_O_3_ also contribute to the improved photocatalytic performance. These traits exemplify the opportunities that exist for atom-scale engineering in heterogeneous CO_2_ photocatalysis, another step towards the vision of the solar CO_2_ refinery.

## Abstract (web summary)

Surface frustrated Lewis pairs (SFLPs) provide a unique class of active sites that enable efficient gas-phase CO_2_ photocatalysis. How to tailor the reactivity of the SFLPs represents a major challenge, which the authors address here by single-site Bi^3+^ ion substitution of the SFLPs.

## Introduction

The increasing energy demands of civil society have accelerated the consumption of coal, oil and natural gas, and associated greenhouse gas emissions. This situation is tipping the delicate balance of CO_2_ in our atmosphere, leading to global warming. To this end, the photocatalytic hydrogenation of CO_2_ into value-added chemicals and fuels has attracted global attention, touted a promising means of achieving a carbon-neutral economy^1–3^. Although materials such as Pd/Nb_2_O_5_^4^, Ru/Al_2_O_3_^5^, LDH nanosheets^6^, and Co-PS@SiO_2_^7^ have been successfully employed as photocatalysts for CO_2_ hydrogenation, a photocatalyst does not currently exist that can meet all the stringent requirements for practical application, including a broad solar response, high conversion efficiency, robust stability and low cost. This renders the design of a practical photocatalyst for CO_2_ hydrogenation a challenge.

Besides catalyst modifications designed to broaden spectral response and improve charge transfer efficiency, it is also important to accelerate conversion rates of H_2_ or CO_2_ on specially designed surface sites to boost photon and energy efficiency. Recently, surface frustrated Lewis pairs (SFLPs) have shown a propensity towards H_2_ dissociation and CO_2_ activation, a key enabler for many catalytic reactions, including hydrogenation, hydroamination and CO_2_ reduction^8–10^. Generally speaking, SFLPs comprise proximal Lewis acidic and Lewis basic sites providing synergetic activation of reactant molecules. For example, SFLPs sites in the In_2_O_3–*x*_(OH)_y_ photocatalyst, comprises a coordinately unsaturated Lewis acidic In atom, proximal to an oxygen vacancy and an adjacent Lewis basic hydroxide group, enable the heterolysis of H_2_ and reaction with CO_2_ to form either CO or CH_3_OH^11–13^. As well, SFLPs involving coordinately unsaturated surface cobalt sites adjacent to surface hydroxides in the CoGeO_2_(OH)_2_ photocatalyst form CH_4_ from H_2_O and CO_2_^14^. In addition, SFLPs in oxygen vacancy laden CeO_2_ bearing SFLPs catalyze the hydrogenation of alkenes and alkynes^15^. All of these cases utilize oxygen vacancies and hydroxides to engineer the catalytic activity of SFLPs. How to tailor the reactivity of the SFLPs themselves is rarely mentioned.

Indium sesquioxide (In_2_O_3_) is proving to be a promising catalyst for the thermal hydrogenation of CO_2_ to CH_3_OH or CO^16–18^. Experimental and computational studies of CO_2_ hydrogenation over oxygen vacancy laden In_2_O_3_ revealed that methanol formation was favored over the reverse water gas shift (RWGS) reaction^19,20^. Methanol production was remarkably enhanced when In_2_O_3_ was supported on ZrO_2_ arising from electronic support effects^21^. A bifunctional catalyst composed of partially reduced In_2_O_3_ supported on HZSM-5 could convert CO_2_ directly into gasoline-range hydrocarbons with a 78.6% selectivity due to the synergistic effects of these two components^22^. By controlling the degree of non-stoichiometry in In_2_O_3–*x*_, a black indium oxide catalyst, which utilized the entire solar spectrum, facilitated the photothermal RWGS reaction under ambient conditions with 100% selectivity^23^. Tailoring the electronic properties of In_2_O_3_ can also be achieved via replacement of an indium atom in the lattice with a H_2_ spillover palladium atom, although the rarity and cost of palladium could prove an issue for its practical implementation^24^.

Bismuth, regarded as a “green” element, has a long and fascinating history^25^. The Incas in sixteenth century South America, made corrosion resistant bronzes for their knives by mixing bismuth with tin^26^. Paracelsus in fifteenth century Germany, recognized bismuth as a non-toxic brother to lead^27^. Since, it is finding myriad eco-friendly uses from cosmetics and personal care products to medicine and lubricants. Most recently it has proved to be a serious contender for replacing toxic lead halide perovskite materials in solar cells with non-toxic bismuth oxyiodide, retaining a comparable energy conversion efficiency of 22%^28^. Contextually, bismuth materials with layered structures and visible light absorption properties, exemplified by BiOX (X=Cl, Br, I), Bi_2_MO_6_ (M=Mo, W), BiVO_4_ and Bi_2_S_3_, behave as photocatalysts to be applied in dye degradation, water and carbon dioxide reduction^29^.

Described herein, we developed a facile solvothermal route to achieve atom-precise substitution of Bi^3+^ for In^3+^ sites in In_2_O_3_ and realize the tailor of the reactivity of SFLPs, as well as the electronic properties of In_2_O_3_. To amplify, by substituting cheaper and safer bismuth for indium in UV absorbing In_2_O_3_, one can create the broad-spectrum UV–Vis light absorber Bi_*x*_In_2–*x*_O_3_. Significantly, single-site Bi^3+^ substitution for In^3+^ provides strong Lewis acidic/basic Bi^3+^–O^2−^ pairs that enhance CO_2_ adsorption and activation, while Bi 6s^2^ lone-pair electrons create mid-gap energy states. Atom-precise lattice engineering of this kind, boosts the reactivity of SFLPs and the harvesting efficiency of solar photons by Bi_*x*_In_2–*x*_O_3_ compared to In_2_O_3_, which enables 1000 times photoactivity enhancement of the RWGS reaction together with a noticeable increase in the production of solar methanol.

## Results

### Structural characterizations of single-site Bi_*x*_In_2–*x*_O_3_

**Fig. 1:**
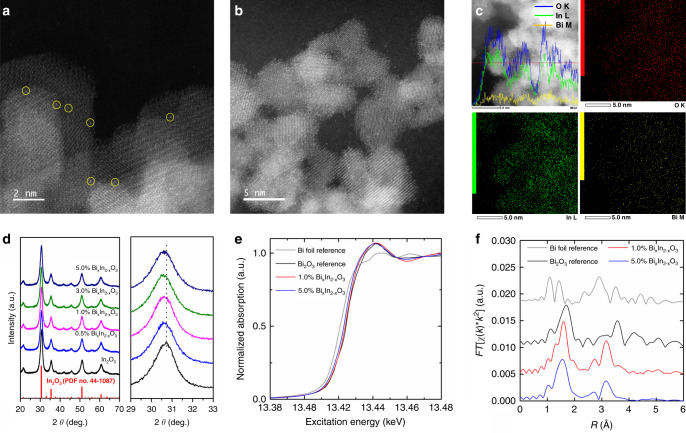
Structural characterizations of single-site Bi^3+^ substituted Bi_*x*_In_2–*x*_O_3_. **a** Aberration-corrected STEM image of 1.0% Bi_*x*_In_2–*x*_O_3_ nanocrystals. **b** Aberration-corrected STEM image of 5.0% Bi_*x*_In_2–*x*_O_3_ nanocrystals. **c** EDS mapping profiles of 5.0% Bi_*x*_In_2–*x*_O_3_ along the indicated red line. **d** PXRD patterns of Bi_*x*_In_2–*x*_O_3_ nanocrystals and pristine In_2_O_3_. **e** Normalized Bi L_3_-edge XANES spectra of 1.0% and 5.0% Bi_*x*_In_2–*x*_O_3_, as well as Bi foil and Bi_2_O_3_ references. **f** k^3^-Weighted Bi L_3_-edge Fourier-transform EXAFS spectra of 1.0% and 5.0% Bi_*x*_In_2–*x*_O_3,_ as well as Bi foil and Bi_2_O_3_ references.

Bi_*x*_In_2–*x*_O_3_ nanocrystals were prepared via a one-step solvothermal route, in which the molar ratio of Bi could be controlled by adjusting the concentration of Bi(NO_3_)_3_ and In(NO_3_)_3_ precursors. The mole percent Bi content of each sample in the Bi_*x*_In_2–*x*_O_3_ series of nanocrystals was determined using inductively coupled plasma mass spectrometry (ICP-MS, Supplementary Table 1). Transmission electron microscopy (TEM) shows that the pristine In_2_O_3_ nanocrystals are flower-like agglomerates of small nanocrystals with an average size of 3.7 nm (Supplementary Fig. 1). Bi^3+^ substitution results in similarly sized Bi_*x*_In_2–*x*_O_3_ nanocrystals (3.5 nm) that show lattice fringes with a spacing of 2.92 Å, corresponding to the (222) plane of bcc In_2_O_3_ (Supplementary Fig. 2). The obtained selected area electron diffraction pattern shown no evidence of any metallic Bi or Bi_2_O_3_. Most significantly, spherical aberration-corrected scanning transmission electron microscopy (STEM) images provide an insightful and distinct result, in which atomically dispersed single-site Bi atoms are revealed under these high-resolution imaging conditions as bright dots (Fig. 1a, b). Energy-dispersive X-ray spectroscopy (EDS) line scans and elemental mapping (Fig. 1c and Supplementary Fig. 3) provided further evidence for the homogeneous distribution of elemental Bi in these Bi_*x*_In_2–*x*_O_3_ nanocrystals.

The phase structure of the obtained Bi_*x*_In_2–*x*_O_3_ nanocrystals was studied by powder X-ray diffraction (PXRD, Fig. 1d). All the Bi_*x*_In_2–*x*_O_3_ nanocrystals displayed nearly identical XRD patterns diagnostic of face-centered cubic In_2_O_3_ except that the diffraction peaks were shifted to lower 2*θ* values relative to those of pristine In_2_O_3_. This result indicates In^3+^ were isomorphously substituted by Bi^3+^, which has a larger ionic radius than In^3+^ (i.e., 0.96 Å versus 0.81 Å). This conclusion is supported by the In 3d peaks in the corresponding X-ray photoelectron spectroscopy (XPS) spectra (Supplementary Fig. 4a), which exhibited a gradual positive energy shift for Bi_*x*_In_2–*x*_O_3_ nanocrystals relative to pure In_2_O_3_, that is attributed to the higher electronegativity of Bi^3+^ compared to In^3+^. The spin–orbit coupled doublet of Bi 4f XPS peaks at 158.6 eV and 163.9 eV define the oxidation state of bismuth as Bi^3+^ rather than Bi^0^ (Supplementary Fig. 4b), following the isomorphous substitution of In^3+^ by Bi^3+^. The electron paramagnetic resonance (EPR) spectra of Bi_*x*_In_2–*x*_O_3_ nanocrystals at both room temperature and 77 K revealed the absence of paramagnetic species, thereby providing further evidence for the lack of Bi^0^ (Supplementary Fig. 5). Possibly EPR for semiconductors with high populations of [O]v occupied by electrons either does not exist or the existence of electronically degenerate ground states with fast electron relaxation and line broadening creates EPR silence. Maybe also the [O]v are devoid of trapped electrons or are doubly filled and hence diamagnetic and EPR silent. Further studies, such as 4 probe Van Der Paaw electrical conductivity measurements, are necessary to fully elucidate the conduction electron model.

Synchrotron radiation-based X-ray absorption spectroscopy (XAS) was further used to obtain information regarding the local structural environment of these distributed Bi sites. The Bi L_3_^−^edge X-ray absorption near-edge structure (XANES) spectra in Fig. 1e reveal visible similarities between the 1.0% and 5.0% Bi_*x*_In_2–*x*_O_3_ sample spectra and that of the Bi_2_O_3_ reference. These similarities are to be expected, given that Bi atoms in both Bi_2_O_3_ and Bi_*x*_In_2–*x*_O_3_ lattices are expected to be octahedrally coordinated by oxygen atoms, leading to similarities in their structural and electronic properties. The Fourier-transformed Bi L_3_-edge extended X-ray absorption fine structure (EXAFS) spectra are presented in Fig. 1f. The similar positions of the Bi–O peak positions suggest that these bonds lengths in the Bi_*x*_In_2–*x*_O_3_ samples are similar to those in the Bi_2_O_3_ reference. In stark contrast, though, the observed Bi–M peaks appear at distinctly different positions in the Bi_*x*_In_2–*x*_O_3_ spectra, revealing a distinct structural difference relative to the Bi_2_O_3_ reference. In order to more accurately quantify these differences in bond length and structure, the spectra were also fitted to extract key structural parameter values (Supplementary Fig. 6 and Supplementary Table 2). The resulting Bi–In bond lengths in the Bi_*x*_In_2–*x*_O_3_ samples are shorter than those found in the pristine Bi_2_O_3_ lattice, though slightly longer than those observed in the pristine In_2_O_3_ lattice. Relatively larger Debye-Waller coefficient values for the Bi–O peaks in Bi_*x*_In_2–*x*_O_3_ samples were also observed, reflecting a broader range of constituent Bi–O bond lengths.

### CO_2_ hydrogenation performance

**Fig. 2:**
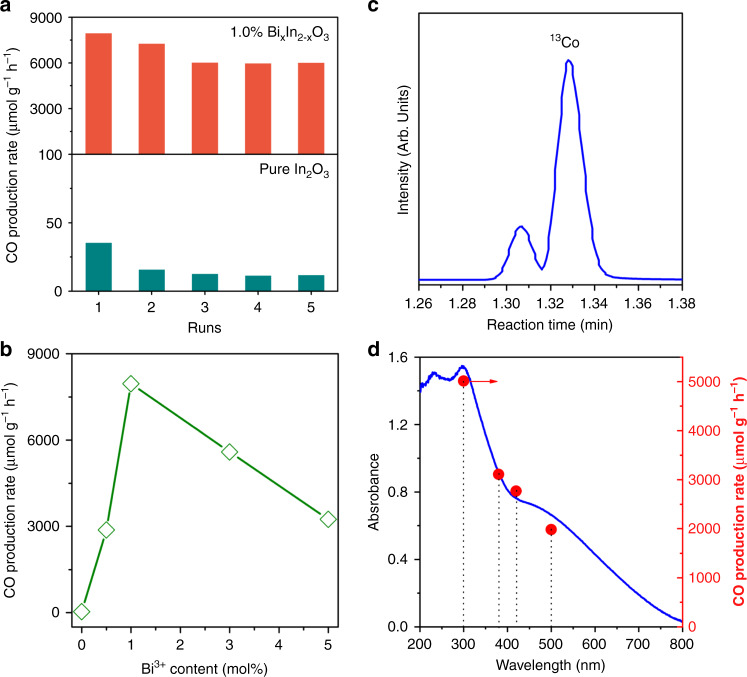
Photocatalytic performance in the batch reactor. **a** CO rate of pristine In_2_O_3_ (down) and 1.0% Bi_*x*_In_2–*x*_O_3_ nanocrystals (up) in catalyzing hydrogenation of CO_2_ under illumination. **b** CO rate as a function of Bi^3+^ content for various Bi_*x*_In_2–*x*_O_3_ nanocrystals. **c** GC-MS plot of ^13^CO produced from ^13^CO_2_ over 1.0% Bi_*x*_In_2–*x*_O_3_ nanocrystals. **d** CO rate as a function of absorption cutoff filter wavelength for 1.0% Bi_*x*_In_2–*x*_O_3_ nanocrystals.

The photocatalytic CO_2_ hydrogenation activity of Bi_*x*_In_2–*x*_O_3_ nanocrystals was evaluated in a batch reactor under simulated solar light irradiation and using a 1:1 ratio of CO_2_ and H_2_ gases. In these experiments, the RWGS reaction (i.e., CO_2_ + H_2_ → CO + H_2_O) led to CO being the sole product detected. The CO production rates revealed that 1.0% Bi_*x*_In_2–*x*_O_3_ was more active than In_2_O_3_ by approximately three orders of magnitude (Fig. 2a), with an impressive peak rate of 8000 μmol g^−1 ^ h^−1^ for its first run, as compared to just 35 μmol g^−1^ h^−1^ for pristine In_2_O_3_. The estimated turnover frequency (TOF) of In_2_O_3_ and 1.0% Bi_*x*_In_2–*x*_O_3_ is 0.42 h^−1^ and 93.6 h^−1^, respectively (Supplementary Note), implying that substituting Bi atoms into In_2_O_3_ nanocrystals significantly enhanced the photocatalytic activity towards CO_2_ hydrogenation. Remarkably, this boost in catalytic activity was much more dramatic than that observed in analogous hydroxylated systems (i.e., Bi_*x*_In_2–*x*_O_3_(OH)_*y*_)^30^, thereby suggesting a distinct and potent mechanism of catalytic activity enhancement in Bi_*x*_In_2–*x*_O_3_. Furthermore, such a CO production rate is much higher than some of the most active noble metal decorated photocatalysts (Supplementary Table 3). In addition, the 1.0% Bi_*x*_In_2–*x*_O_3_ was very stable, exhibiting a CO production rate that was still roughly 600 times greater than the 11 μmol g^−1^ h^−1^ exhibited by pristine In_2_O_3_ under the same experimental conditions. In both cases, the CO production rate was observed to decrease over the course of five consecutive runs.

The actual bulk reaction temperature for In_2_O_3_ and Bi_*x*_In_2–*x*_O_3_ tested by infrared camera is about 70 and 115 °C (Supplementary Fig. 7), respectively. The relatively low thermal energy supplied by solar light indicates the limited contribution of photothermal effect on the photocatalytic RWGS reaction. As an endothermic reaction, the rate of the RWGS is expected increase quite rapidly as a function of temperature. According to the Arrhenius Law, the rate should approximately double for every 10 °C increase in temperature. Thus, we would expect the reaction rate to increase by a factor of ~22.5, assuming that the bulk temperature of the catalyst accurately reflects the temperature of the catalytically active sites. Based on the rate of pristine In_2_O_3_ (i.e., 35 μmol g^−1^ h^−1^), this would result in a rate increase to about 788 μmol  g^−1^ h^−1^ and account for about 10 % of the observed activity increase, thereby suggesting the existence of a significant photochemical effect. Notably, the marked increase in CO production rate upon substituting single-site Bi atoms into In_2_O_3_ was highly dependent on the concentration of Bi atoms (Fig. 2b). Meanwhile, isotopically labeled ^13^CO_2_ experiment confirmed that CO was the unequivocal product from photocatalytic CO_2_ hydrogenation (Fig. 2c).

We also investigated the dependence of the CO production rate on the wavelength of light, to demonstrate the CO_2_ hydrogenation proceeds mainly through a photocatalytic process. As seen in the action spectra shown in Fig. 2d and Supplementary Fig. 8, the production rate of CO monotonically decreased with longer wavelengths of the light, which correlates with the optical absorption spectrum of pristine In_2_O_3_ and Bi_*x*_In_2–*x*_O_3_ catalysts. It should also be mentioned that the CO production rate remained at about 2000 μmol g^−1^ h^−1^ on 1.0% Bi_*x*_In_2–*x*_O_3_, even when a 500 nm cutoff filter was applied, thus implying that Bi_*x*_In_2–*x*_O_3_ nanocrystals can function as broadband, green photocatalysis for harvesting solar energy.

**Fig. 3:**
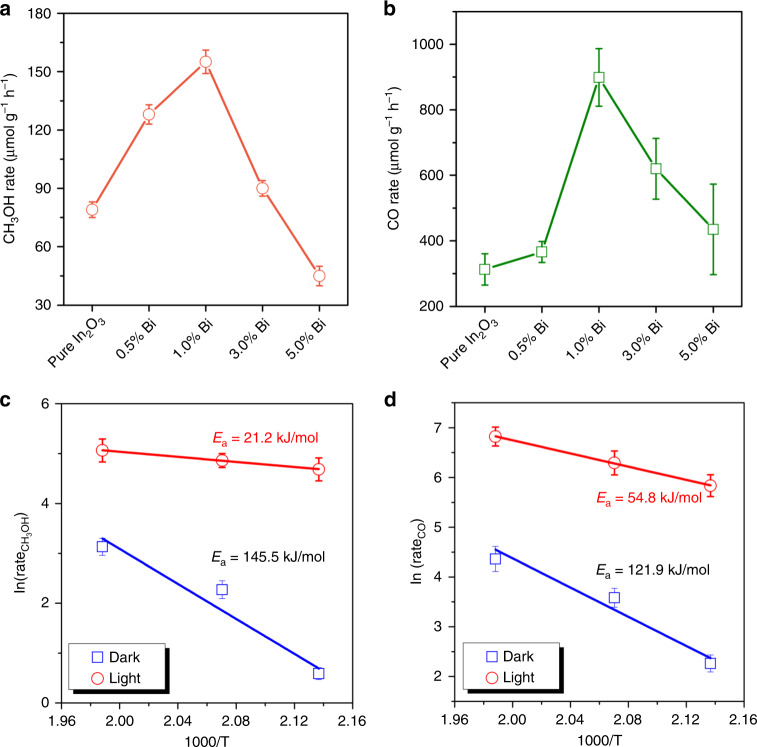
Catalytic performance in the flow reactor with and without light irradiation. **a** CH_3_OH production rates as a function of Bi^3+^ content under light irradiation and at 230 °C. **b** CO production rates as a function of Bi^3+^ content under light irradiation and at 230 °C. **c** Arrhenius plots for CH_3_OH production rates of 1.0% Bi_*x*_In_2–*x*_O_3_ with and without solar irradiation. **d** Arrhenius plots for CO production rates of 1.0% Bi_*x*_In_2–*x*_O_3_ with and without solar irradiation.

In light of the promising performance of Bi_*x*_In_2–*x*_O_3_ nanocrystals towards gas-phase CO_2_ hydrogenation, this new catalyst was also studied for solar methanol production in a flow reactor at 230 °C, both with and without light irradiation. As shown in Supplementary Fig. 9, all catalysts exhibited similarly low CO and CH_3_OH production rates under purely thermal conditions; however, a remarkable enhancement in the production rates of CO and CH_3_OH was obtained on changing from dark to light conditions. In this case, pristine In_2_O_3_ exhibited CO and CH_3_OH production rates of 312 and 82 μmol g^−1 ^h^−1^, respectively (Fig. 3a, b). In comparison, the single-site Bi_*x*_In_2–*x*_O_3_ samples exhibited much better activities for both CO and CH_3_OH production, with 1.0% Bi_*x*_In_2–*x*_O_3_ exhibiting the highest CO and CH_3_OH production rates of 918 μmol g^−1^ h^−1^ and 158 μmol g^−1^ h^−1^, respectively. Overall, the measured activities of the Bi_*x*_In_2–*x*_O_3_ samples were highly dependent on Bi content and showed a volcano-shaped trend. The volcano trend of the activity towards CO_2_ hydrogenation versus the extent of In^3+^ substitution by the larger, more electronegative, 6s^2^ stereochemically active lone-pair containing Bi^3+^, can be attributed to a subtle interplay of numerous and competing intertwined properties: chemical effects (e.g., influence of surface Lewis acidity and basicity of In-O-In, In-O-Bi, Bi-O-Bi sites on CO_2_-H_2_ adsorption, activation, reaction processes) and physical effects (e.g., photogenerated electron and hole charge-separation and charge-trapping by bismuth and oxygen vacancy mid-gap states).

The CH_3_OH and CO production rates of Bi_*x*_In_2–*x*_O_3_ nanocrystals showed negligible deactivation, even after 50 h of continuous testing under light irradiation at 230 °C (Supplementary Fig. 10a), suggesting their excellent catalytic stability. The recorded XRD patterns, TEM images and XPS spectra (Supplementary Fig. 10b–d) for the spent Bi_*x*_In_2–*x*_O_3_ photocatalysts after 50 h of reaction demonstrate that, except for a slight increase in particle size, the phase and oxidation states of the nanocrystals were well maintained, confirming their favorable structural stability.

To obtain more information on the origin of the activity enhancement under flow reaction conditions, activity tests were also conducted at lower reaction temperatures, beginning where products can be observed (130, 195, and 210 °C), with and without light irradiation (Supplementary Fig. 11). The drastic activity difference between dark and light conditions lend further support confirmed the contribution of the photochemical effect on CO and CH_3_OH production. Moreover, based on the Arrhenius plots for 1.0% Bi_*x*_In_2–*x*_O_3_, the apparent activation energy for the CO and CH_3_OH photochemical processes are much lower than the thermochemical ones (Fig. 3c, d), reflecting the solar advantage for the excited-state reaction pathway relative to the ground state pathway^31^.

### Photocatalytic reaction pathway

**Fig. 4:**
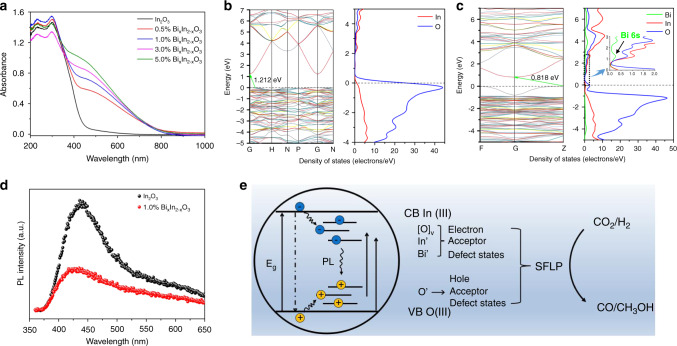
Electronic structures of Bi_*x*_In_2–*x*_O_3_. **a** Diffuse reflectance spectra of pristine In_2_O_3_ and various Bi_*x*_In_2–*x*_O_3_ nanocrystals. **b** The simulated band structure and DOS plots of pristine In_2_O_3_. **c** The simulated band structure and DOS plots of Bi_*x*_In_2–*x*_O_3_ nanocrystals. **d** Room-temperature PL spectra of pristine In_2_O_3_ and 1.0% Bi_*x*_In_2–*x*_O_3_ nanocrystals using an excitation wavelength of 325 nm. **e** Schematic illustrating charge carrier recombination pathways on surface defects states (SFLPs) and enabling CO_2_ hydrogenation reactions.

The photocatalytic CO_2_ hydrogenation reaction involves photon-absorption, electron-hole separation, and CO_2_ adsorption/activation processes. The first two steps are closely related to the intrinsic nature of the photocatalyst, while the third is highly dependent on the gas-solid interface. A significant red shift occurs in the absorption edge of the UV–Vis spectra for all Bi_*x*_In_2–*x*_O_3_ samples (Fig. 4a), along with an enhanced tail above 440 nm, which grows with Bi^3+^ content and is accompanied by a change in color from cream to rust (Supplementary Fig. 12). This is consistent with the simulated band structures that show the introduction of Bi^3+^ can leads to the formation of mid-gap states in the bandgap of In_2_O_3_ (Fig. 4b, c). The total density of states (DOS) and partial density of states (PDOS) (Supplementary Fig. 13) can further reveal that the substitution of Bi at the In site induces mid-gap energy states with Bi 6s states below the conduction band edge of In_2_O_3_ and is consistent with the reported results in this paper^32–34^.

To understand the photogenerated charge transfer mechanism, the room-temperature photoluminescence (PL) spectra of pristine In_2_O_3_ and 1.0% Bi_*x*_In_2–*x*_O_3_ nanocrystals are shown in Fig. 4d. The pristine In_2_O_3_ nanocrystals exhibited a strong green emission peak centered at ca. 440 nm, originating from the radiative recombination of photo-excited electrons trapped in mid-gap oxygen vacancy states with photogenerated holes in the valence band^35^. The existence of oxygen vacancies is further evidenced by the O 1 s core level XPS spectra (Supplementary Fig. 14). In contrast, the incorporation of single-site Bi into In_2_O_3_ leads to weakening and broadening of the PL emission peak. This can be explained in two ways. One is that the substitutional Bi^3+^ slightly decreases the concentration of oxygen vacancies (Supplementary Fig. 15), which would decrease the intensity due to electron-hole radiative recombination. Alternatively, the substituted Bi^3+^ states lying below the conduction band of In_2_O_3_ could also act as traps capturing photo-excited electrons and inhibiting electron-hole recombination emission, resulting in lower PL emission. Thus, as illustrated in Fig. 4e, the substituted Bi^3+^ sites (denoted as Bi’), oxygen vacancies [O], coordinately unsaturated indium In’ sites and oxygen O’ sites, exist as mid-gap defect states (comprising surface frustrated Lewis pairs, SFLPs) in the bandgap of Bi_*x*_In_2–*x*_O_3_, can function as traps for photogenerated electrons and holes enabling the reaction between CO_2_/H_2_^36,37^. This results in the quenching of steady-state PL emission as well as a slight shortening of the average fluorescence lifetime from 120 to 110 ps, probed by time-resolved PL spectroscopy (Supplementary Fig. 16). Albeit small, this reduction of the fluorescence lifetime, suggests that, relative to In_2_O_3_, single-site Bi atoms can increase the occurrence of competitive non-radiative relaxation processes in Bi_*x*_In_2–*x*_O_3_^38–40^.

**Fig. 5:**
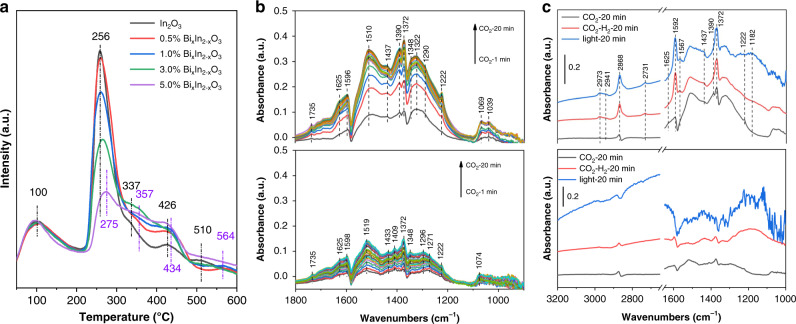
In situ DRIFTS experiments of CO_2_ adsorption and CO_2_ hydrogenation. **a** CO_2_-TPD profiles. **b** In situ DRIFTS spectra for the adsorption of CO_2_ on 1.0% Bi_*x*_In_2–*x*_O_3_ nanocrystals (up) and pure In_2_O_3_ (down). **c** In situ DRIFTS spectra of surface species under reaction conditions on 1.0% Bi_*x*_In_2–*x*_O_3_ nanocrystals (up) and pure In_2_O_3_ (down).

Apart from its effect on the electronic structure and charge transfer, single-site Bi^3+^ substitution is also expected to strengthen the adsorption-bonding-activating ability of Bi_*x*_In_2–*x*_O_3_ toward CO_2_. The textural structure of Bi_*x*_In_2–*x*_O_3_ including surface area, pore volume and pore size show obvious improvements and could favor the adsorption of CO_2_ reactants (Supplementary Table 4 and Supplementary Fig. 17). With respect to the surface chemistry, CO_2_ can bond through its carbon atom and oxygen atoms to either the surface oxygen atoms, metal sites, or directly with the oxygen vacancies of metal oxides (Supplementary Fig. 18)^41–43^. To investigate the effect of Bi^3+^ substitution on the interaction between CO_2_ and Bi_*x*_In_2–*x*_O_3_ or pristine In_2_O_3_ nanocrystals, CO_2_ temperature-programmed desorption (CO_2_-TPD) measurements were initially performed. As shown in Fig. 5a, one broad desorption peak at around 100 °C, corresponding to physically adsorbed CO_2_, is observed for all Bi_*x*_In_2–*x*_O_3_ nanocrystals and pristine In_2_O_3_. A significant desorption peak is clearly observed at 256 °C for pristine In_2_O_3_ and can be attributed to the chemical desorption of CO_2_ that is binding with oxygen vacancies to form bent CO_2_^δ−^ species^44^. Since Bi^3+^ substitution results in fewer oxygen vacancies, this peak intensity gradually decreases with increased Bi^3+^ doping of Bi_*x*_In_2–*x*_O_3_ nanocrystals and shifts slightly to higher temperatures (as high as 275 °C for 5.0 % Bi_*x*_In_2–*x*_O_3_), implying that the binding strength of CO_2_ and oxygen vacancies is remarkably enhanced. Moreover, weak desorption peaks at higher temperatures (300 to 600 °C) were clearly observed for In_2_O_3_, and can be assigned to the decomposition of surface HCO_3_^−^ and CO_3_^2−^ species^45^. After single-site Bi^3+^ substitution, typical desorption peaks can also be clearly identified and show a slight shift to higher temperatures, again indicating that these surface species are binding more strongly to the surface.

In situ diffuse reflectance infrared Fourier-transform spectroscopy (DRIFTS) experiments were further carried out to identify surface species. Figure 5b show the transient evolution of the surface species during CO_2_ adsorption over 1.0% Bi_*x*_In_2–*x*_O_3_ nanocrystals. The bands at 1510 and 1372 cm^−1^ are assigned to the asymmetric and symmetric OCO stretching modes of monodentate carbonates (*m*-CO_3_^2−^). The features at 1549 and 1330 cm^−1^ are attributed to the asymmetric and symmetric OCO stretching modes of bidentate carbonates (*b*-CO_3_^2−^). The bent CO_2_^δ−^ species adsorbed at oxygen vacancy sites can be identified by two bands at 1596 and 1348 cm^−1^, corresponding to the asymmetric and symmetric stretching modes, respectively. The appearance of bands at 1625, 1437, 1390, and 1222 cm^−1^ indicates the formation of bicarbonate species (HCO_3_^−^). Moreover, a small amount of a linearly adsorbed CO_2_ species with bands appearing between 1000 and 1100 cm^−1^ can also be observed. Thus, via DRIFTS measurements, all the surface species observed during CO_2_-TPD measurements, CO_3_^2−^, HCO_3_^−^ and CO_2_^δ−^, were observed and identified on Bi_*x*_In_2–*x*_O_3_ nanocrystals. All these surface species can be observed on pristine In_2_O_3_; however, the peak intensities of these species are weaker than that on Bi_*x*_In_2–*x*_O_3_ nanocrystals, suggesting the improved CO_2_ adsorption-bonding-activating capacity after Bi^3+^ substitution.

Density functional theory (DFT) slab calculations were further carried out to unravel the promotion effect of Bi^3+^ substitution on In_2_O_3_. The perfect In_2_O_3_ (110) surface was initially selected as it has proven to be most thermodynamically stable^20,46^. The defective In_2_O_3_ (110) surface with an oxygen vacancy at the O_4_ site was then created owing to the more favorable ability for CO_2_ activation and hydrogenation^19^. Following then, we examined the possibility of Bi^3+^ substitution at the In site.

**Fig. 6:**
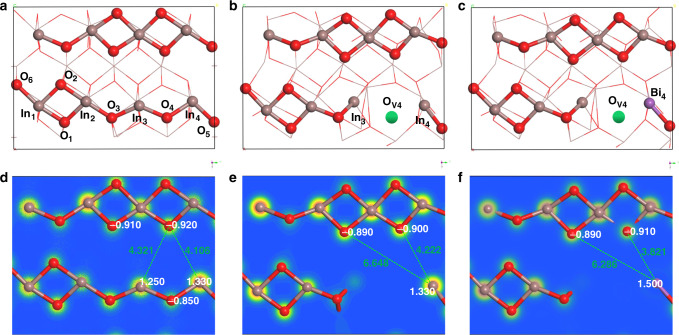
Schematic images of the reactivity of SFLPs in single-site Bi^3+^ substituted Bi_*x*_In_2*–x*_O_3_. **a** Optimized structure of perfect In_2_O_3_ (110). **b** Optimized structure of defective In_2_O_3_ (110) with one oxygen vacancy. **c** Optimized structure of Bi_*x*_In_2–*x*_O_3_ (110) with Bi^3+^ substitution. **d** Electron-density isosurface of perfect In_2_O_3_ (110). **e** Electron-density isosurface of defective In_2_O_3_ (110) with one oxygen vacancy. **f** Electron-density isosurface of Bi_*x*_In_2–*x*_O_3_ (110) with Bi^3+^ substitution.

As shown in Fig. 6a, the perfect In_2_O_3_ (110) surface consists of chains of In and O atoms, with the numbering In and O atoms along the chain as repeating unit. The surface In and O in the chain are adjacent and contiguous to each other, forming a classic Lewis acid-base adjunct, whereas the unbonded In_3_ and In_4_ in the chain and O in the top layer show a distance of 4.106 and 4.312 Å, respectively, which may deliver SFLPs-like activity (Fig. 6d). However, the electronic interactions between In_3_ or In_4_ and its neighboring O_4_ will block the function of In_3_-O or In_4_-O pairs. Therefore, the removal of oxygen atom at the O_4_ site is the prerequisite to construct a pair of unbonded Lewis acid and base sites. When the O_4_ atom is removed, two In atoms (In_3_ and In_4_) are coordinatively unsaturated and one oxygen vacancy (O_V4_) is produced (Fig. 6b). However, in this case, only one In atom (In_4_) locates at the surface while the other one (In_3_) moves to the inner atomic layer. The surface In_4_ atom is found to be surrounded by two adjacent oxygen atoms, of which the In_4_-O with a distance of 4.222 Å can construct a SFLPs site (Fig. 6e). We further investigated the effect of Bi^3+^ substitution at In_4_ site on the configuration and charge population (Fig. 6c, f). As compared to In_4_-O configuration, Bi_4_-O shows a shorter distance (3.821 Å) but can still fall in the domain of solid SFLPs. On the other hand, the Bader charge calculations show that the related Lewis acid Bi^3+^ and Lewis base O^2−^ involve atomic local charges of +1.500 e and −0.910 e, respectively, which is higher than that of the In^3+^ and O^2−^ pair (+1.300 e and −0.900 e). The larger charge difference between the Lewis acid and Lewis base pairs in the Bi_*x*_In_2–*x*_O_3_ compared with that of defective In_2_O_3_ would form more active Lewis acid-base pairs than the In_2_O_3_ pair can muster, and therefore could deliver a higher capability to activate CO_2_ molecules, consistent well with the DRIFTs and CO_2_-TPD results.

In the photo-excited state of a SFLPs system, the Lewis acidity and Lewis basicity have been shown to increase as compared with the ground state, thereby facilitating the photochemical CO_2_ hydrogenation, with a decrease in activation energy. To get more insight into the improved activity from Bi^3+^ substitution, in situ DRIFTS experiments were further performed under reaction conditions to detect the reaction intermediates and uncover the photocatalytic pathway in the CO_2_ hydrogenation process. As shown in Fig. 5c, when the Bi_*x*_In_2–*x*_O_3_ nanocrystals were exposed to the mixture of CO_2_ and H_2_ gases, bidentate formate (*HCOO), methoxy (*H_3_CO) and carboxylate (*CO_2_)^47^, were the three principal intermediates observed from the transformation of bicarbonate and carbonate species, as evidenced by the decrease and disappearance of characteristic bands at 1510, 1390, and 1222 cm^−1^. The *HCOO species can be linked to fingerprint modes at 2973 and 2731 cm^−1^, which correspond to a combination of the CH bending and OCO stretching modes^47,48^. The bands at 1592 and 1370 cm^−1^ can be assigned to the asymmetric and symmetric OCO stretching modes while that at 2868 cm^−1^ is attributed to the CH stretching mode of the same species^47,49,50^. The *H_3_CO species is signaled by diagnostic modes at 2941 and 2838 cm^−1^ that are assigned to the CH_3_ stretching modes and the band at 1182 cm^−1^ is attributed to the CO stretching mode of bridged methoxide species^47,48,51^. In addition to *HCOO and *H_3_CO, *CO_2_ species were also observed, with bands at 1567 and 1379 cm^−1^ that can be associated with the OCO stretching modes. Under light irradiation, all intermediates showed an increase in band intensity (Supplementary Fig. 19), thereby confirming the photochemical effect of CO_2_ hydrogenation, and is consistent with the activity results. From these DRIFT results, CO_2_ hydrogenation over Bi_*x*_In_2–*x*_O_3_ may proceed via two major reaction pathways featured by both formate intermediate and CO intermediate, which has been well established for the In_2_O_3-*x*_(OH)_*y*_ systems^12^. In the case of pristine In_2_O_3_ (Fig. 5c and Supplementary Fig. 20), *HCOO and *H_3_CO species of virtually insignificant intensity were observed for CO_2_ hydrogenation, and light irradiation resulted in much noisier peaks. This further indicates the moderate catalytic performance of pristine In_2_O_3_ and the significant promotion effect resulting from single-site Bi^3+^ substitution. To corroborate these experimental observations, free energy profiles for CO_2_ hydrogenation via the proposed RWGS pathway over Bi_*x*_In_2–*x*_O_3_ and pristine In_2_O_3_ were calculated (Supplementary Fig. 21). It can be seen that the H_2_ dissociation into H* on pristine In_2_O_3_ (defective type with one oxygen vacancy) is the rate-limiting step and endothermic with an activation energy barrier of 1.47 eV. Importantly, compared with pristine In_2_O_3_, the Bi_*x*_In_2–*x*_O_3_ exhibits a negative Δ*G* value of −0.05 eV for the H_2_ dissociation, which implies that the H_2_ dissociation into H* on the surface of Bi_*x*_In_2–*x*_O_3_ is energetically favorable. This result indicates that the single-site Bi^3+^ substituted nanostructure has more active Lewis acid-base pairs than the In_2_O_3_ pair can muster, and therefore can strongly polarize H–H bonds and dissociate H_2_ molecules into *H. The proceeding hydrogenation reactions of H* with CO_2_ on the surface of In_2_O_3_ and Bi_*x*_In_2–*x*_O_3_ are similar. However, benefiting from the favorable H_2_ dissociation, Bi_*x*_In_2–*x*_O_3_ shows a much-lowered reaction energy profile for CO and H_2_O formation than pristine In_2_O_3_.

## Discussion

In summary, we have demonstrated a one-step solvothermal route towards atom-precise isomorphic substitution of In^3+^ in In_2_O_3_ by Bi^3+^ to generate Bi_*x*_In_2–*x*_O_3_ materials with broad-spectrum UV–Vis absorption. The incorporation of single-site Bi atoms in the In_2_O_3_ host lattice provides strong Lewis acid-base Bi^3+^–O^2−^ pairs to enhance CO_2_ adsorption and activation, resulting in distinctly enhanced reaction rates relative to those observed for pristine In_2_O_3_ and other indium oxide-based catalysts. The Bi 6s^2^ lone pairs create mid-gap energy states, which can increase the harvesting of solar photons and favor the generation and separation of photo-induced charge carriers. Remarkably, single-site Bi^3+^-substituted Bi_*x*_In_2–*x*_O_3_ proves to be a highly efficient and stable photocatalyst, achieving an impressive CO production rate three orders of magnitude greater than that of pristine In_2_O_3_, with notable photoactivity towards solar methanol. In addition to increased activity catalytic sites, the greening of indium oxide by single-site bismuth atom substitution represents a new approach to CO_2_ photocatalyst engineering and is a further step towards the vision of a solar CO_2_ refinery.

## Methods

### Synthesis of In_2_O_3_ and Bi_*x*_In_2–*x*_O_3_

Pristine In_2_O_3_ nanocrystals were prepared via a simple solvothermal route. In a typical synthesis, 0.3 g of In(NO_3_)_3_•4.5H_2_O was dissolved in 17 mL anhydrous dimethylformamide solution. After stirring for 30 min, the obtained homogeneous solution was transferred into a Teflon-lined stainless steel autoclave and then heated at 150 °C for 24 h. After being cooled to room temperature, the light-yellow product was collected through centrifugation, washed with ethanol and water, and finally dried at 60 °C in vacuum. Bi^3+^-substituted In_2_O_3_ nanocrystals were prepared using the same method employed for pristine In_2_O_3_, except that various amounts of Bi(NO_3_)_3_•5H_2_O were added to the indium solution prior to solvothermal reaction.

### Material characterizations

The content of Bi in Bi_*x*_In_2–*x*_O_3_ was determined using an inductively coupled plasma mass spectroscopy (ICP-MS) instrument (Optima 7300 DV). Powder X-ray diffraction (PXRD) was performed on a Bruker D2-Phaser X-ray diffractometer, using Cu Kα radiation at 30 kV. X-ray photoelectron spectroscopy (XPS) was performed using a PerkinElmer Phi 5500 ESCA spectrometer in an ultrahigh vacuum chamber with a base pressure of 1 × 10^−9^ Torr. The spectrometer used an Al Kα X-ray source operating at 15 kV and 27 mA. The samples were coated onto carbon tape prior to analysis and all results were calibrated to C1s 284.5 eV. EPR spectra were obtained at room temperature and 77 K using a Bruker A-300-EPR X-band spectrometer. Transmission electron microscopy (TEM) measurements were conducted using a JEM–2010 microscope working at 200 kV. The double spherical aberration-corrected scanning transmission electron microscope (STEM) images were obtained on an FEI Themis Z instrument. X-ray absorption spectra were collected at the BL14W beamline of the Shanghai Synchrotron Radiation Facility (SSRF). The storage ring of the SSRF was operated at 3.5 GeV with a stable current of 200 mA. Using a Si(111) double-crystal monochromator, the data collection was carried out in fluorescence mode using Lytle detector. All spectra were collected under ambient conditions. Diffuse reflectance spectra (DRS) of the powders were obtained for dry-pressed disk samples using a Cary 500 Scan Spectrophotometer (Varian, USA) over a range of 200 to 800 nm. Barium sulfate (BaSO_4_) was used as a reflectance standard. Room-temperature photoluminescence (PL) spectra were measured on an FL/FS 920 (Edinburgh Instruments) system equipped with a 450 W Xe arc lamp as the excitation source and a red sensitive Peltier element-cooled Hamamatsu R2658 PMT as the detector. Time-resolved fluorescence decay spectra were recorded on the Delta Pro (HORIBA instruments) using a 357 nm laser as the excitation source. BET surface area analyses were performed on an ASAP2020 M apparatus (Micromeritics Instrument Corp., USA) with the samples degassing in vacuum at 110 °C for 10 h and then measuring at 77 K. The CO_2_ temperature-programmed desorption (CO_2_-TPD) measurements were carried out on AutoChem II 2920 Version. The density functional theory (DFT) calculations were performed using the Cambridge Sequential Total Energy Package (CASTEP) computational codes. During the geometry optimization, lattice parameters and atomic positions were optimized simultaneously. Based on the experimental data, we replaced one In with Bi in the cell as the In_15_BiO_24_ model and deleted one of the oxygen atom that were coordinating with Bi to establish one In_15_BiO_23_(O_Vacancy_) model. For calculating the electronic structures and density of states, the geometry optimization of In_2_O_3_ and In_15_BiO_23_(O_Vacancy_) were calculated by the PBE method within Generalized Gradient-corrected Approximation (GGA), using the exchange-correlation potential. The Vanderbilt ultrasoft pseudopotential with a cutoff energy of 380 eV was used to ensure the precision of the results. Brillouin zone integration was represented using the K-point sampling scheme of 3 ×  3 × 3 Monkhorst–Pack scheme. The convergence tolerance for geometry optimization was selected with the differences in total energy (5.0 × 10^−6^ eV/atom), the maximal ionic Hellmann–Feynman force (1.0 × 10^−2^ eV Å^−1^), the stress tensor (2.0 × 10^−2^ GPa), and the maximal displacement (5.0 × 10^−4^ Å).

### Gas-phase CO_2_ hydrogenation tests

Batch reactions were conducted in a custom-built 1.5 mL stainless steel batch reactor with a fused-silica viewport sealed with Viton O-rings. The reactor with ∼4.5 mg of catalyst on a borosilicate film support was evacuated using an Alcatel dry pump prior to being purged with the reactant high-purity H_2_ reactant gas. After purging the reactor, it was filled with a 1:1 stoichiometric mixture of H_2_ (99.9995%) and CO_2_ (99.999%) until the total pressure reached 30 psi. The reactor was irradiated with a 300 W Xe lamp for a duration of 1 h without external heating. Product gases were analyzed using flame ionization and thermal conductivity detectors installed in a SRI-8610 gas chromatograph equipped with 3 in. Mole Sieve 13a and 6 in. Haysep D column. Isotopically labeled tracing experiments were performed using ^13^CO_2_ (99.9 at%, Sigma-Aldrich). Isotope distributions in the product gases were measured using an Agilent 7890A gas chromatograph-mass spectrometer with a 60 m GS-carbon plot column, leading to the mass spectrometer. Flow experiments were carried out in a fixed-bed tubular reactor with ∼10 mg of catalyst material being packed into a quartz tube and immobilized at both ends with quartz wool. The quartz tube had an inner diameter of 2 mm with a wall thickness of 0.5 mm, and was placed into a groove carved out into a copper block. An OMEGA temperature controller was attached to two heating cartridges inserted into the copper block and a thermocouple was inserted into the quartz tube contacting the catalyst but covered by the quartz wool. A 300 W Xe arc lamp illuminated the catalyst plug at a measured intensity of 2 W cm^−2^. CO_2_ and H_2_ were flowed through with a 1:3 ratio (1 sccm CO_2_, 3 sccm H_2_). The amounts of CO and CH_3_OH produced were determined using gas chromatography-mass spectrometry (GC-MS, 7890B and 5977A, Agilent) using a He carrier gas.

### In situ DRIFT studies

The in situ DRIFTS measurements were performed to detect the surface intermediates over pristine In_2_O_3_ and Bi_*x*_In_2–*x*_O_3_ nanocrystals under reaction conditions. The spectra were collected using a Fourier-transform infrared spectroscopy spectrometer (Thermo, Nicolet 6700) equipped with an MCT detector. Before measurement, the catalyst was purged with He at 250 °C for 2 h. The catalyst was subsequently cooled down to 230 °C. The background spectrum with a resolution of 4 cm^−1^ was obtained at 230 °C in He flow. Then the catalyst was exposed to a mixture of CO_2_, H_2_, and He (1 sccm CO_2_, 3 sccm H_2_, and 16 sccm He, respectively) in dark and light conditions for different times. The in situ DRIFT spectra were recorded by collecting 32 scans at 4 cm^−1^ resolutions.

## Supplementary information

Supplementary Information
