# Supplementary material for: Bismuth atom tailoring of indium oxide surface frustrated Lewis pairs boosts heterogeneous CO2 photocatalytic hydrogenation
Source: Nat Commun. 2020 Nov 30;11:6095. doi: 10.1038/s41467-020-19997-y (PMC7705729; doi:10.1038/s41467-020-19997-y)
Supplement: Supplementary file 1 — Supplementary Information [file 41467_2020_19997_MOESM1_ESM.pdf]

Supporting Information

Bismuth Atom Tailoring of Indium Oxide Surface Frustrated Lewis Pairs Boosts Heterogeneous CO_2 Photocatalytic Hydrogenation

Yan *et al*.

Supplementary Table 1. The molar contents of Bi in the series of $Bi_xIn_{2-x}O_3$ nanocrystals determined by ICP-MS.

Catalyst	Molar content of Bi (%)
0.5% $Bi_xIn_{2-x}O_3$	0.43
1.0% $Bi_xIn_{2-x}O_3$	0.89
3.0% $Bi_xIn_{2-x}O_3$	2.35
5.0% $Bi_xIn_{2-x}O_3$	4.84

Supplementary Table 2. Fitting results of Bi L_3-edge and In K-edge FT-EXAFS data.

Sample	Path	CN	R (Å^2)	σ^2 (Å^2)	ΔE_0 (eV)
Bi_2O_3	Bi-O	2.8(4)	2.169(9)	0.005(1)	1.5(7)
	Bi-O	2.7(4)	2.52(3)	0.022(9)	
	Bi-Bi	3.8(9)	3.590(8)	0.006(1)	
1.0% $Bi_xIn_{2-x}O_3$	Bi-O	5(1)	2.21(1)	0.016(4)	-7(1)
	Bi-In	4(1)	3.42(1)	0.007(1)	
5.0% $Bi_xIn_{2-x}O_3$	Bi-O	4.4(6)	2.18(1)	0.014(3)	-8.6(8)
	Bi-In	2.9(7)	3.42(1)	0.009(1)	
In_2O_3	In-O	6.1(3)	2.170(3)	0.0061(9)	-0.4(4)
	In-In	4.9(6)	3.359(3)	0.0031(6)	
	In-In	3.6(9)	3.843(4)	0.003(1)	

CN = coordination numbers of Bi and In atoms; R = bond length; σ^2 = the Debye−Waller factor coefficient.

Supplementary Table 3. The compared CO production rate of different catalysts for photocatalytic CO_2 hydrogenation.

Catalyst	Feeds (CO_2+H_2)	Light Source	T (°C)	CO rate (μmol·g^{-1}·h^{-1})	Ref.
$In_2O_{3-x}(OH)_y$ superstructures	1:1	1000 W Hortilux Blue metal halide bulb	-	1.2	1
$Bi_zIn_{2-z}O_{3-x}(OH)_y$	1:1	1000 W Hortilux Blue metal halide bulb	150	1.32	2
Pd/Nb_2O_5	1:1	300 W Xe lamp	-	1800	3
$Pd@H_yWO_{3-x}$	1:1	300 W Xe lamp	250	3000	4
$Pt/NaTaO_3$	1:1	300 W UV-enhanced Xe lamp	-	139.1	5
Pd@SiNS	1:1	300 W Xe lamp	170	10	6
$In_2O_{3-x}(OH)_y/Nb_2O_5$	1:1	300 W Xe lamp	-	1400	7
**$Bi_xIn_{2-x}O_3$**	**1:1**	**300 W Xe lamp**	**-**	**8000**	**This work**

Supplementary Table 4. Specific surface area of pristine In_2O_3 and $Bi_xIn_{2-x}O_3$ nanocrystals.

Catalyst	Surface Area (m^2/g)	Pore Volume (cm^3/g)	Pore Size (nm)
Pristine In_2O_3	104.0	0.145087	12.1
0.5% $Bi_xIn_{2-x}O_3$	118.6	0.279663	11.7
1.0% $Bi_xIn_{2-x}O_3$	147.8	0.304572	9.8
3.0% $Bi_xIn_{2-x}O_3$	157.3	0.331772	9.1
5.0% $Bi_xIn_{2-x}O_3$	152.4	0.288860	7.9

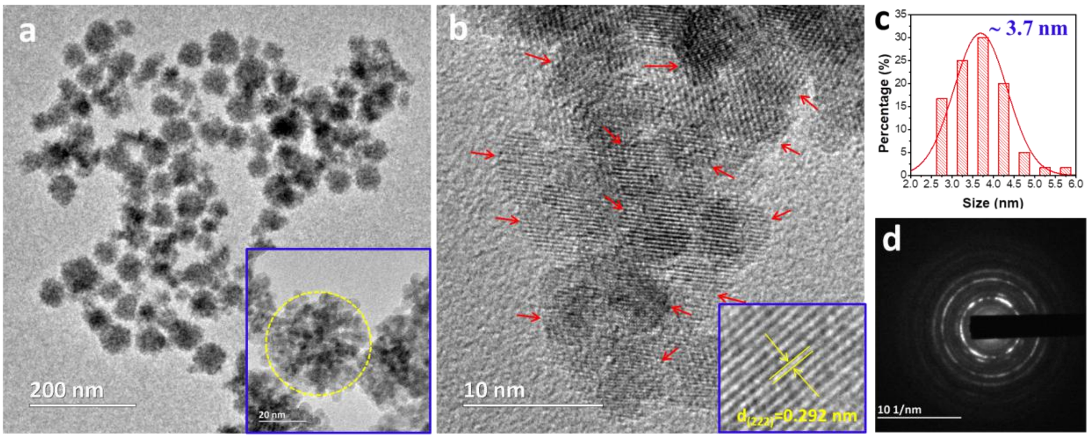

Supplementary Figure 1. (a) TEM, (b) HRTEM, (c) size distribution, and (d) SAED of pristine In_2O_3. The highlighted yellow ring in (a) shows one flower-like agglomerate. The red arrows in (b) represent the single nanocrystals, and the inset in (b) shows the lattice fringe with a spacing of 2.92 Å**.**

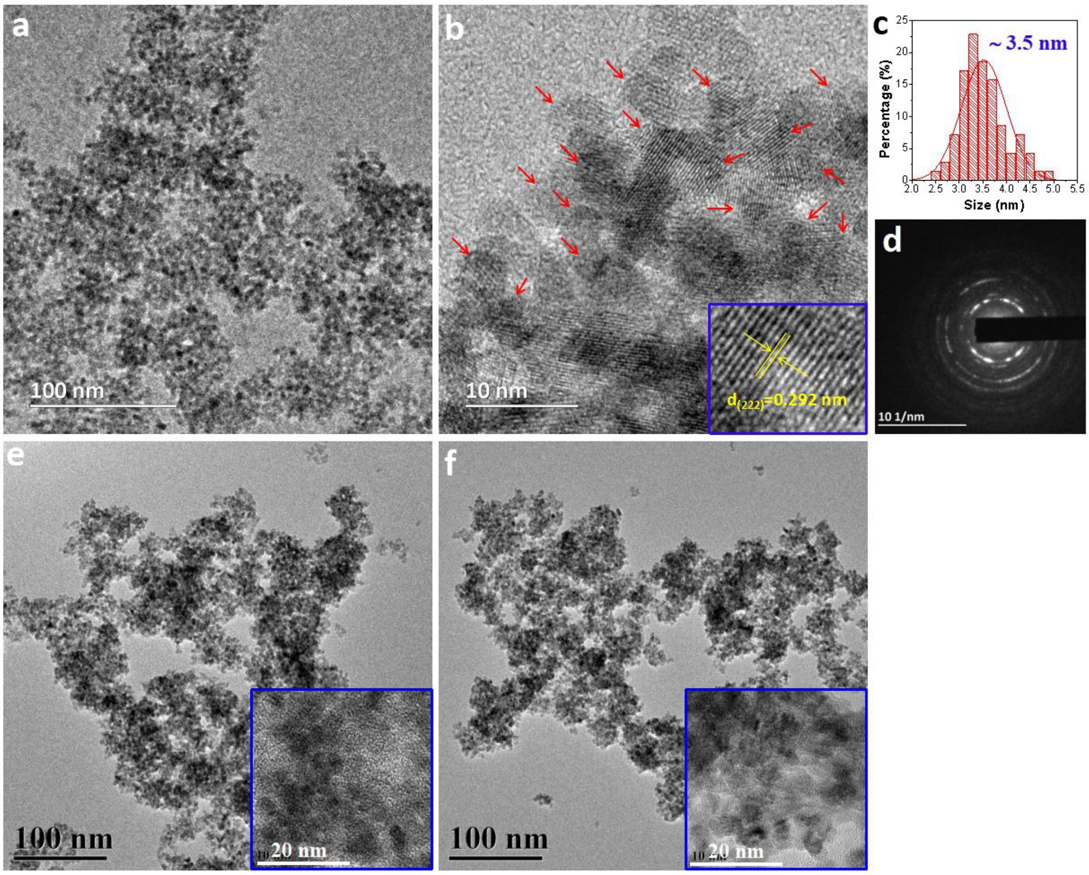

Supplementary Figure 2. (a) TEM, (b) HRTEM, (c) size distribution, and (d) SAED of 1.0% $Bi_xIn_{2-x}O_3$ sample. (e) TEM and HRTEM of 3.0% $Bi_xIn_{2-x}O_3$ sample. (f) TEM and HRTEM of 5.0% $Bi_xIn_{2-x}O_3$ sample.

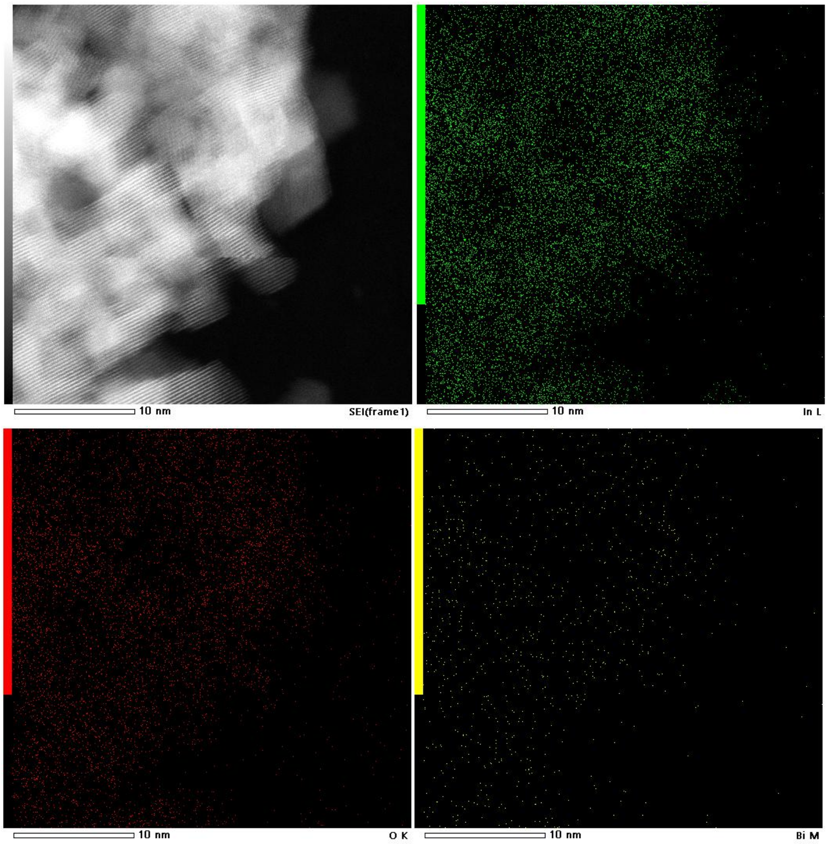

Supplementary Figure 3. Elemental mapping profiles of 1.0% $Bi_xIn_{2-x}O_3$.

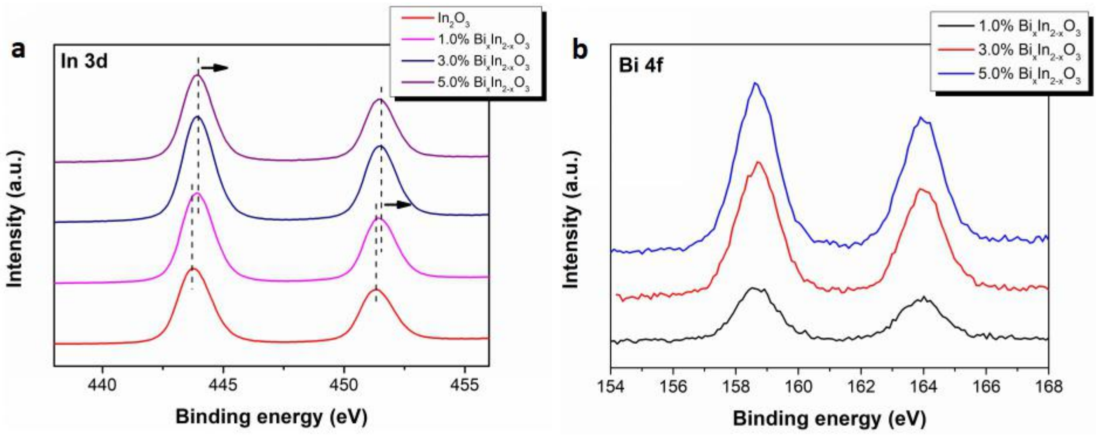

Supplementary Figure 4. (a) In 3d and (b) Bi 4f XPS spectra of pure In_2O_3 and $Bi_xIn_{2-x}O_3$ nanocrystals.

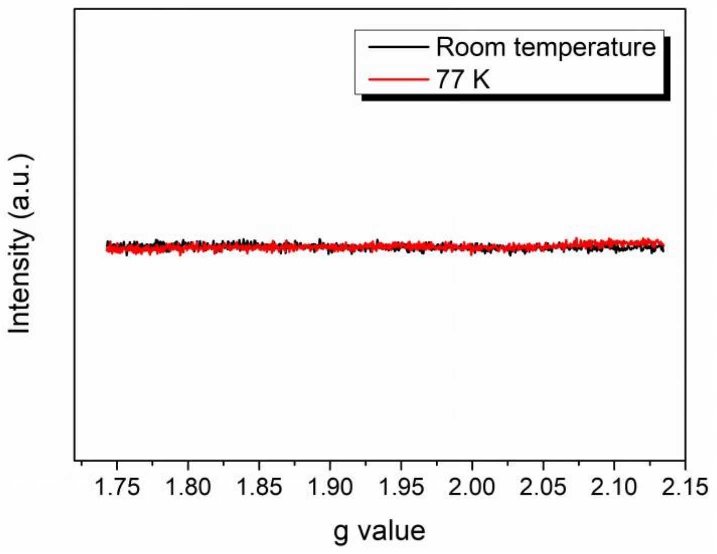

Supplementary Figure 5. EPR spectra of 1.0% $Bi_xIn_{2-x}O_3$ nanocrystals recorded at room temperature and 77 K.

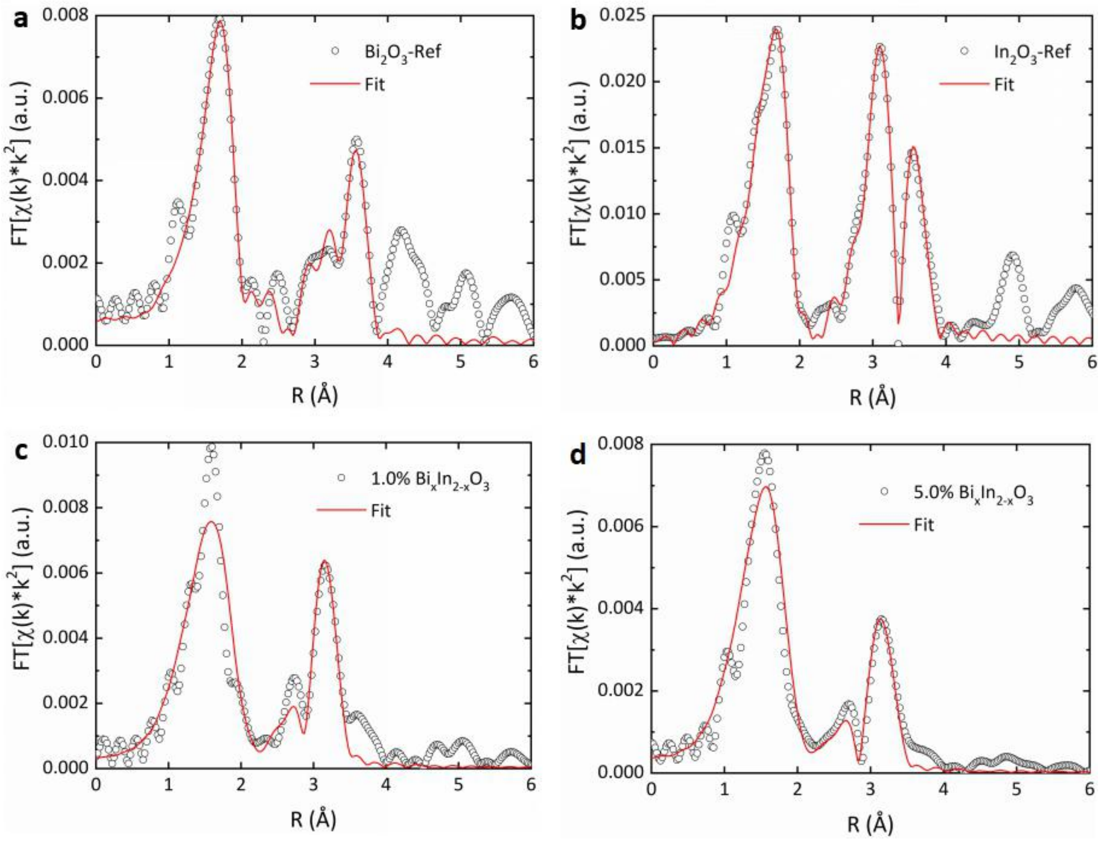

Supplementary Figure 6. Fitted Bi L_3-edge and In K-edge FT-EXAFS spectra from (a) Bi_2O_3 and (b) In_2O_3 reference materials, as well as (c) 1.0% and (d) 5.0% $Bi_xIn_{2-x}O_3$ samples.

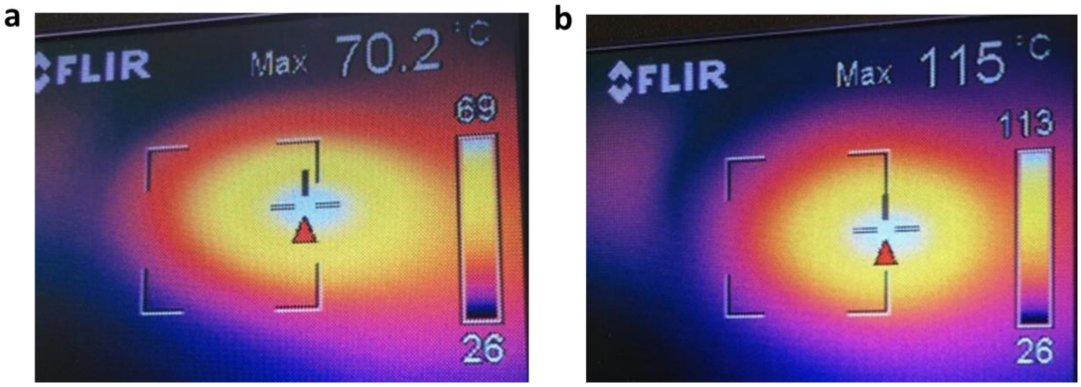

Supplementary Figure 7. The actual reaction temperature tested by IR camera on (a) pristine In_2O_3 and (b) 1.0% $Bi_xIn_{2-x}O_3$ nanocrystals.

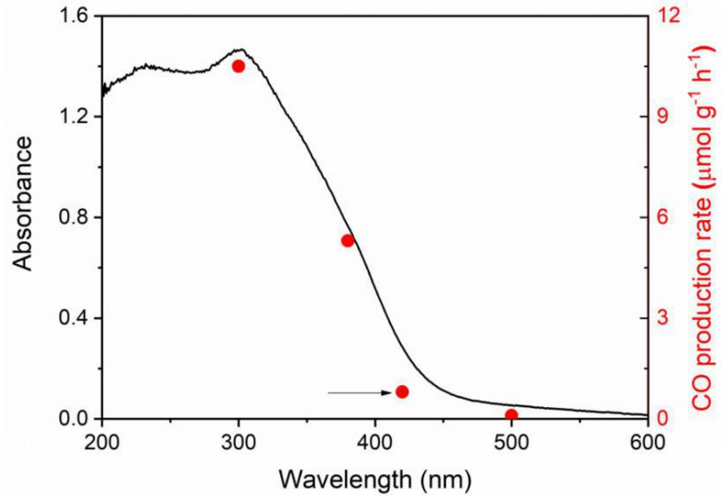

Supplementary Figure 8. CO production rate as a function of absorption cut-off filter wavelength for pure In_2O_3 sample.

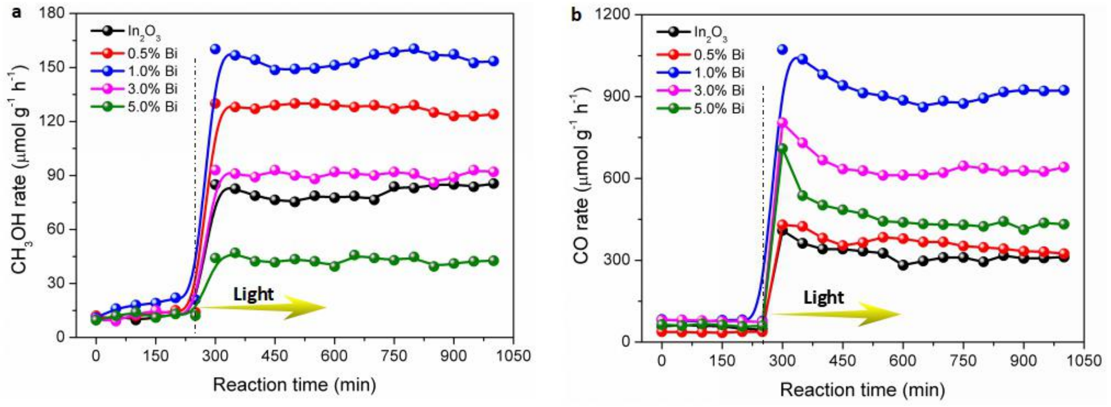

Supplementary Figure 9. (a) CH_3OH production and (b) CO production as function of reaction time on pristine In_2O_3 and $Bi_xIn_{2-x}O_3$ nanocrystals in the flow reactor at 230 °C, with and without light irradiation.

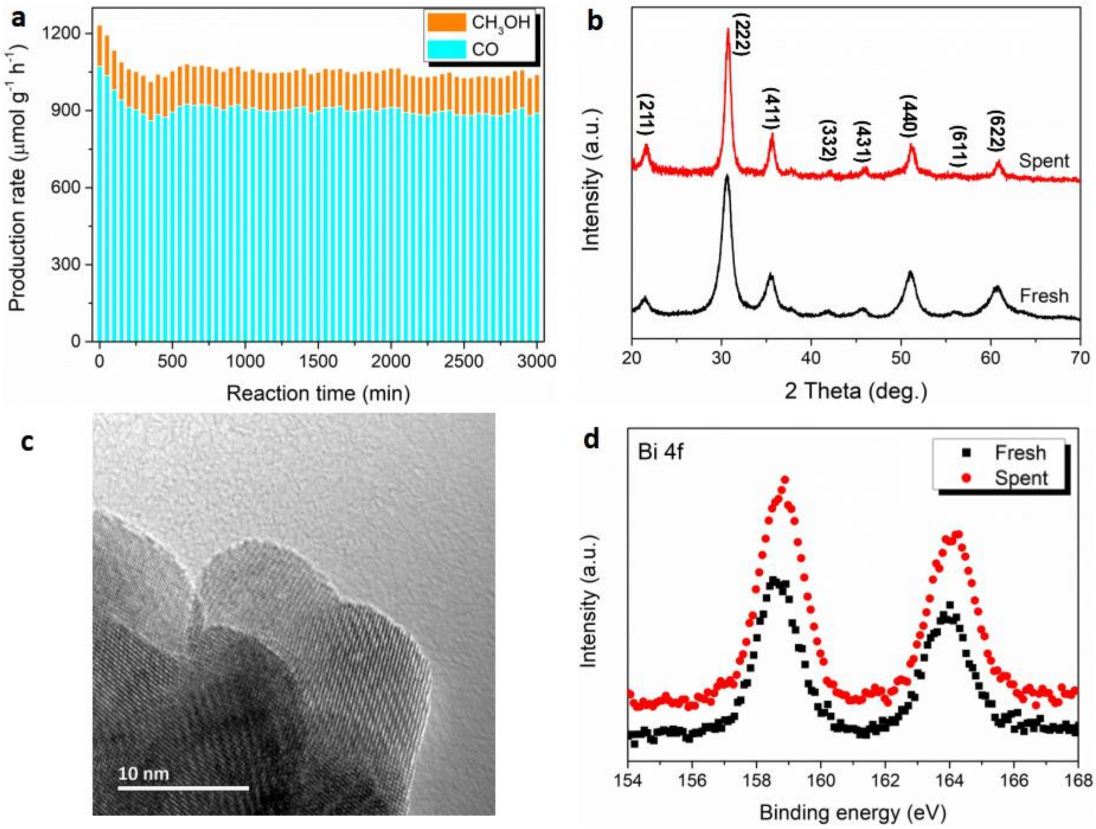

Supplementary Figure 10. (a) Plotted reaction rates demonstrating the long-term (50 h) catalytic stability of 1.0% $Bi_xIn_{2-x}O_3$ nanocrystals during photocatalytic CO_2 hydrogenation. (b) XRD patterns, (c) TEM image, and (d) Bi 4f XPS spectra of fresh and spent (*i.e.,* after 50 h stability testing) 1.0% $Bi_xIn_{2-x}O_3$ nanocrystals.

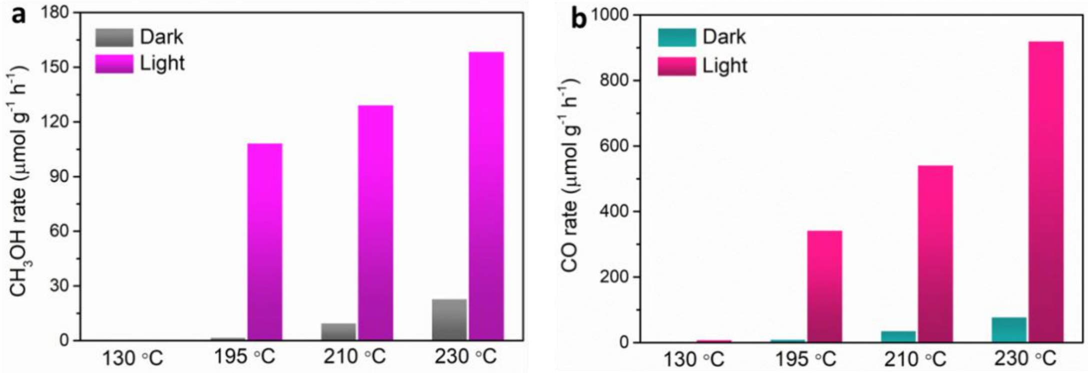

Supplementary Figure 11. (a) CH_3OH production rates and (b) CO production rates of the top-performing catalyst 1.0% $Bi_xIn_{2-x}O_3$ as a function of reaction temperatures.

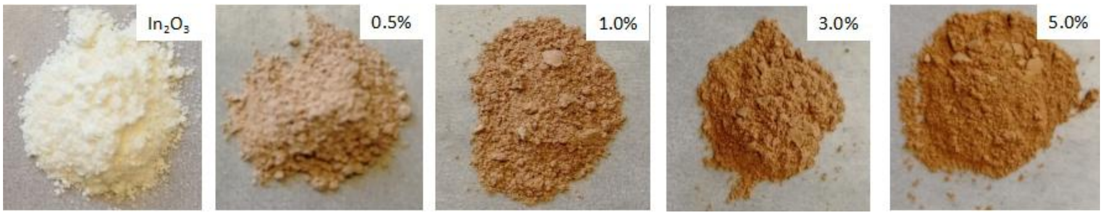

Supplementary Figure 12. The color of pure In_2O_3 and substituted $Bi_xIn_{2-x}O_3$ nanocrystals.

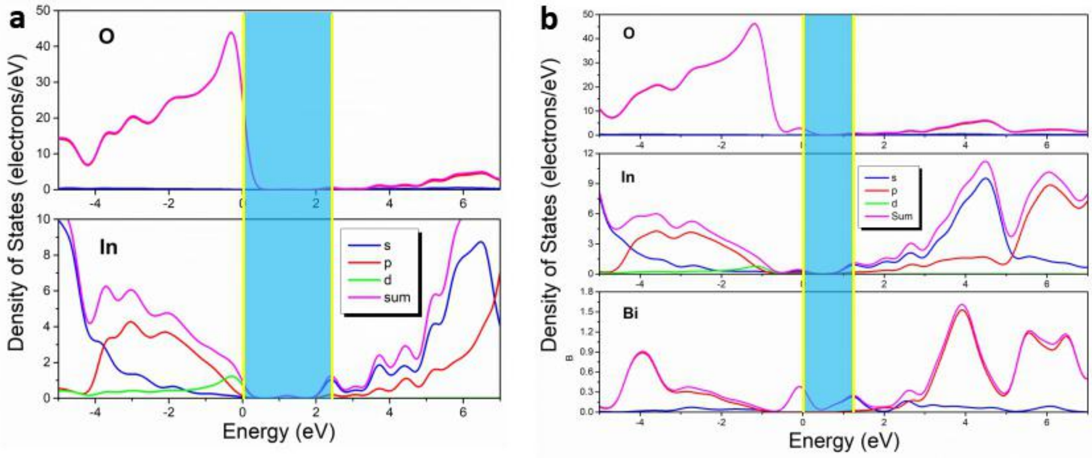

Supplementary Figure 13. Calculated DOS plots for (a) pristine In_2O_3 and (b) $Bi_xIn_{2-x}O_3$ nanocrystals.

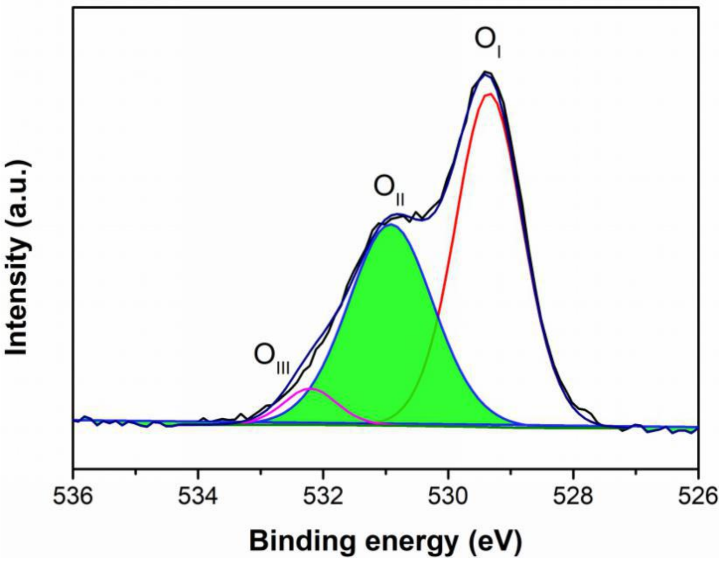

Supplementary Figure 14. High-resolution O 1s core-level XPS spectra of 1.0% $Bi_xIn_{2-x}O_3$ nanocrystals. The O 1s core level XPS spectra could be fitted into three peaks at 529.3 eV, 530.9 eV, and 532.8 eV, which are assigned to oxides (O_I), oxygen vacancies (O_{II}), and hydroxyl groups (O_{III}), respectively.

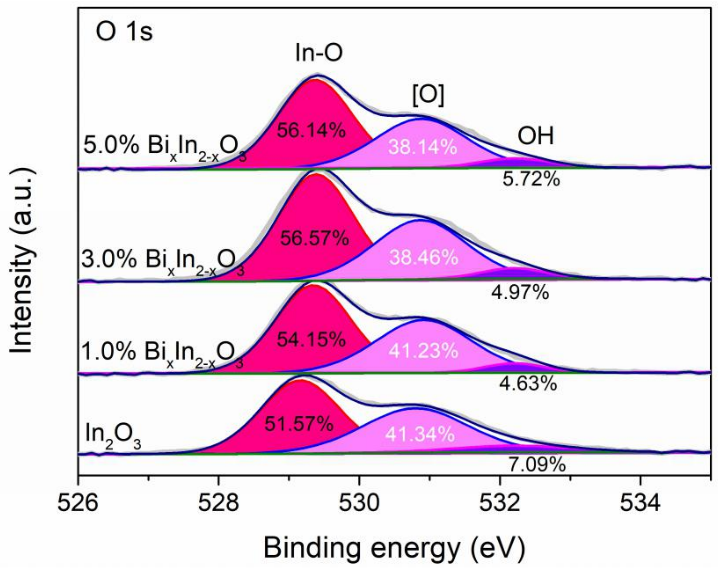

Supplementary Figure 15. High-resolution O 1s core-level XPS spectra of pure In_2O_3 and $Bi_xIn_{2-x}O_3$ nanocrystals.

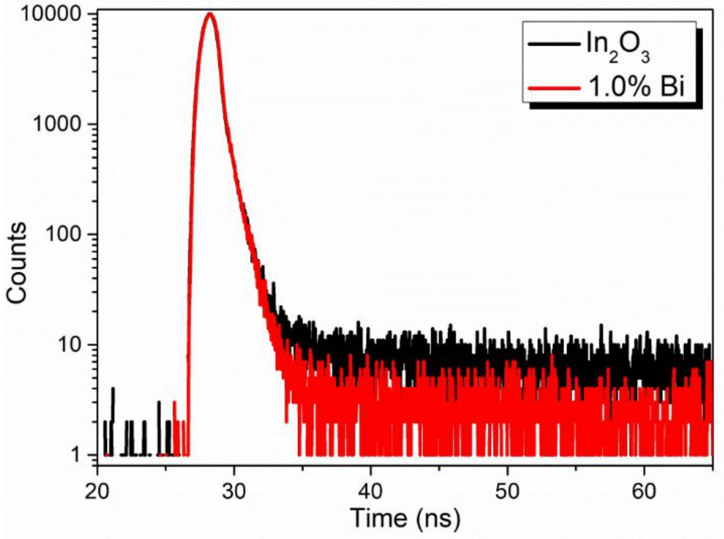

Supplementary Figure 16. Time-resolved PL spectra of pristine In_2O_3 and 1.0% $Bi_xIn_{2-x}O_3$ nanocrystals.

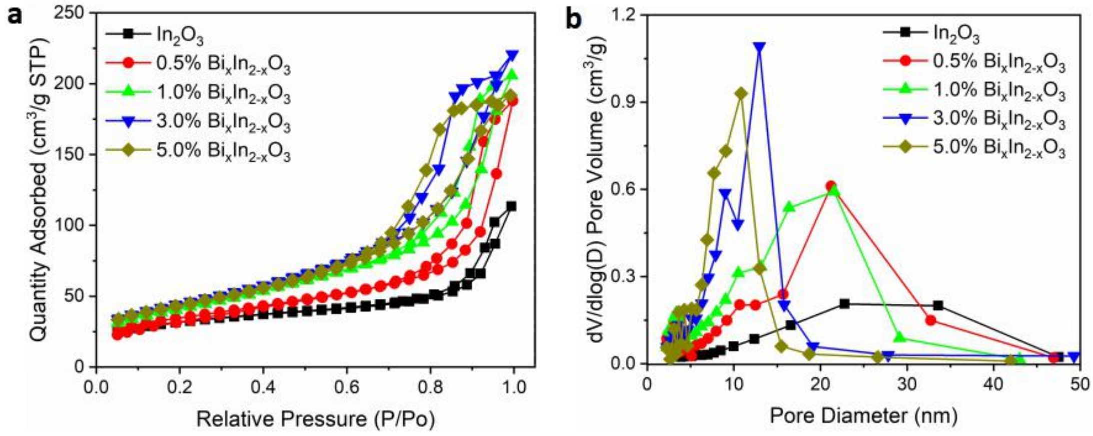

Supplementary Figure 17. N_2 sorption isotherms (a) and pore size distributions (b) of pristine In_2O_3 and $Bi_xIn_{2-x}O_3$ nanocrystals.

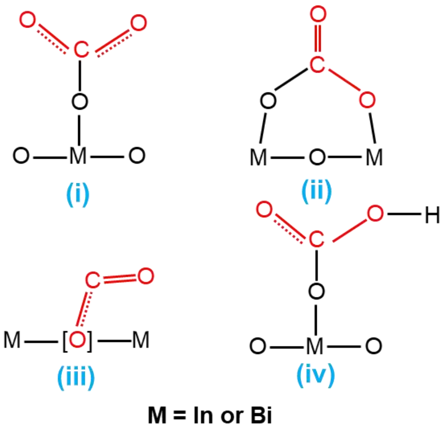

Supplementary Figure 18. Schematic of surface species contributing to the adsorption of CO_2 on $Bi_xIn_{2-x}O_3$ nanocrystals, as exemplified by monodentate carbonate-like species (*m*-CO_3^{2-}), bidentate carbonate-like species (*b*-CO_3^{2-}), bent adsorbed species ($CO_2^{\delta -}$), and bicarbonate-like species (HCO_3^-).

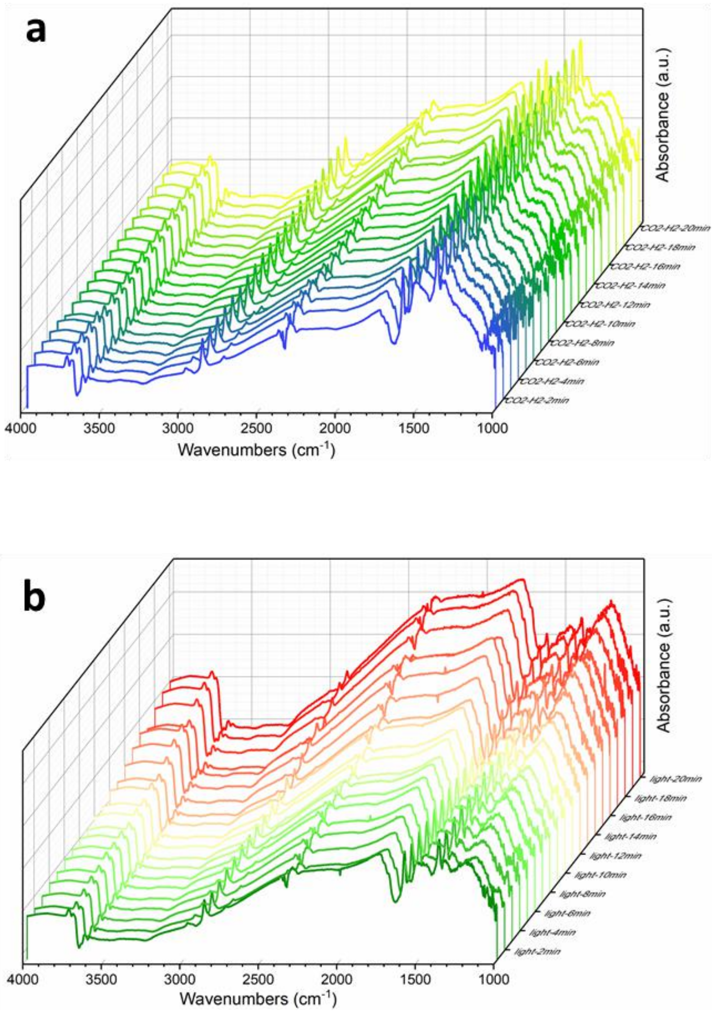

Supplementary Figure 19. *In-situ* DRIFTS spectra for CO_2 hydrogenation under (a) dark and (b) light conditions on 1.0% $Bi_xIn_{2-x}O_3$.

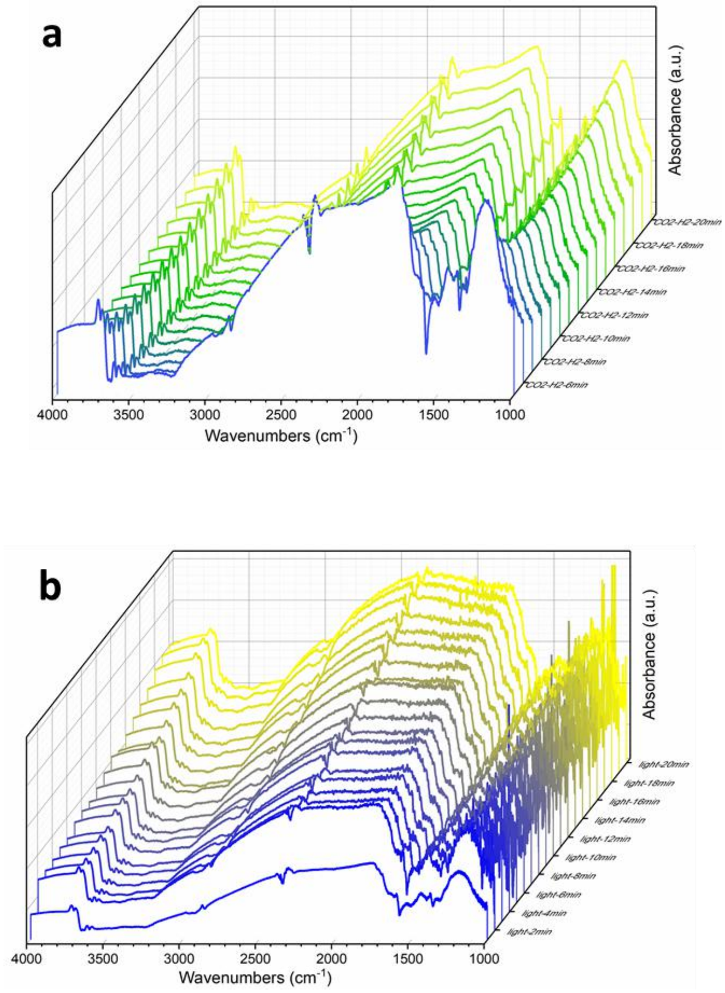

Supplementary Figure 20. *In-situ* DRIFTS spectra for CO_2 hydrogenation under (a) dark and (b) light conditions on pristine In_2O_3.

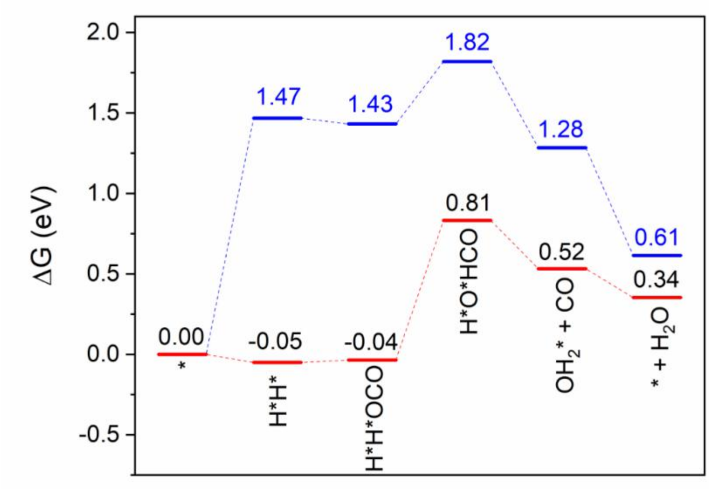

Supplementary Figure 21. Energy profiles for CO_2 hydrogenation via the RWGS pathway on the surface of pristine In_2O_3 (blue line) and $Bi_xIn_{2-x}O_3$ (red line).

Supplementary Note

Calculation of turnover frequency (TOF)

$$\text{TOF} = \frac{\text{number of produced molecues}}{\text{number of active sites * total reaction time}} \quad \textbf{(Supplementary Equation 1)}$$

$N_A = 6.022 \times 10^{17}\ \mu mol^{-1}$

$$\text{Number of produced molecules per hour} = \frac{\text{CO rate} * N_A}{\text{total reaction time}} \quad \textbf{(Supplementary Equation 2)}$$

Total reaction time = 1 h

Number of surface O atoms at exposed facet = $9.23 \times 10^{18}\ m^{-2}$

✧ Pristine In_2O_3 rate: 35.6 μmol $h^{-1}g^{-1}$ → 1.06 μmol $h^{-1}m^{-2}$ (accoring to the surface area: 33.5 m^2g^{-1})

The number of produced molecules per hour for pristine In_2O_3 is $6.38 \times 10^{17}\ m^{-2}h^{-1}$

✧ 1.0% $Bi_xIn_{2-x}O_3$ rate: 7959 μmol $h^{-1}g^{-1}$ → 232.72 μmol $h^{-1}m^{-2}$ (accoring to the surface area: 34.2 m^2g^{-1})

The number of produced molecules per hour for 1.0% $Bi_xIn_{2-x}O_3$ is $1.4 \times 10^{20}\ m^{-2}h^{-1}$

Model 1: Assume all [O] will be used for reaction

Number of active sites = number of [O] = $9.23 \times 10^{18}\ m^{-2}$ x [O] value obtained from XPS (41.34%) = $3.82 \times 10^{18}\ m^{-2}$

$TOF_{In2O3} = 6.38 \times 10^{17}\ m^{-2}h^{-1} / 3.82 \times 10^{18}\ m^{-2} = 0.167\ h^{-1}$

Similarly, $TOF_{1.0\%\ BixIn2-xO3} = 36.8\ h^{-1}$.

Model 2: Assume only extrinsic [O] will be used for reaction

Number of active sites = number of extrinsic [O] = $9.23 \times 10^{18}\ m^{-2}$ x ([O] value obtained from XPS (41.34%) – intrinsic [O] of In_2O_3 (25%)) = $1.51 \times 10^{18}\ m^{-2}$

$TOF_{In2O3} = 6.38 \times 10^{17}\ m^{-2}h^{-1} / 1.51 \times 10^{18}\ m^{-2} = 0.42\ h^{-1}$

As a result, $TOF_{1.0\%\ BixIn2-xO3} = 93.6\ h^{-1}$.

Supplementary References

(1) He, L. et al. Spatial separation of charge carriers in $In_2O_{3-x}(OH)_y$ nanocrystal superstructures for enhanced gas-phase photocatalytic activity. *ACS Nano* **10**, 5578-5586 (2016).

(2) Dong, Y. C. et al. Tailoring surface frustrated Lewis pairs of $In_2O_{3-x}(OH)_y$ for gas-phase heterogeneous photocatalytic reduction of CO_2 by isomorphous substitution of In^{3+} with Bi^{3+}. *Adv. Sci.* **5**. 1700732-1700742 (2018).

(3) Jia, J. et al. Visible and near-Infrared photothermal catalyzed hydrogenation of gaseous CO_2 over nanostructured $Pd@Nb_2O_5$. *Adv. Sci.* **3**, 1600189-1600201 (2016).

(4) Li, Y. F. et al. $Pd@H_yWO_{3-x}$ nanowires efficiently catalyze the CO_2 heterogeneous reduction reaction with a pronounced light effect. *ACS Appl. Mater. Interfaces* **11**, 5610-5615 (2019).

(5) Li, M. et al. Highly efficient and stable photocatalytic reduction of CO_2 to CH_4 over Ru loaded $NaTaO_3$. *Chem. Commun.* **51**, 7645-7648, (2015).

(6) Qian, C. X. et al. Catalytic CO_2 reduction by palladium-decorated silicon-hydride nanosheets. *Nat. Catal.* **2**, 46-54 (2019).

(7) Wang, H. et al. Heterostructure engineering of a reverse water gas shift photocatalyst. *Adv. Sci.* **6**, 1902170-1902175 (2019).
